# Patterning in Placental 11-B Hydroxysteroid Dehydrogenase Methylation According to Prenatal Socioeconomic Adversity

**DOI:** 10.1371/journal.pone.0074691

**Published:** 2013-09-05

**Authors:** Allison A. Appleton, David A. Armstrong, Corina Lesseur, Joyce Lee, James F. Padbury, Barry M. Lester, Carmen J. Marsit

**Affiliations:** 1 Department of Community and Family Medicine, Geisel School of Medicine at Dartmouth, Hanover, New Hampshire, United States of America; 2 Department of Pharmacology and Toxicology, Geisel School of Medicine at Dartmouth, Hanover, New Hampshire, United States of America; 3 Department of Pediatrics, Women and Infants Hospital, Providence, Rhode Island, United States of America; 4 Brown Center for the Study of Children at Risk, Women and Infants Hospital, Providence, Rhode Island, United States of America; King Faisal Specialist Hospital & Research center, Saudi Arabia

## Abstract

**Background:**

Prenatal socioeconomic adversity as an intrauterine exposure is associated with a range of perinatal outcomes although the explanatory mechanisms are not well understood. The development of the fetus can be shaped by the intrauterine environment through alterations in the function of the placenta. In the placenta, the *HSD11B2* gene encodes the 11-beta hydroxysteroid dehydrogenase enzyme, which is responsible for the inactivation of maternal cortisol thereby protecting the developing fetus from this exposure. This gene is regulated by DNA methylation, and this methylation and the expression it controls has been shown to be susceptible to a variety of stressors from the maternal environment. The association of prenatal socioeconomic adversity and placental *HSD11B2* methylation has not been examined. Following a developmental origins of disease framework, prenatal socioeconomic adversity may alter fetal response to the postnatal environment through functional epigenetic alterations in the placenta. Therefore, we hypothesized that prenatal socioeconomic adversity would be associated with less *HSD11B2* methylation.

**Methods and Findings:**

We examined the association between DNA methylation of the *HSD11B2* promoter region in the placenta of 444 healthy term newborn infants and several markers of prenatal socioeconomic adversity: maternal education, poverty, dwelling crowding, tobacco use and cumulative risk. We also examined whether such associations were sex-specific. We found that infants whose mothers experienced the greatest levels of socioeconomic adversity during pregnancy had the lowest extent of placental *HSD11B2* methylation, particularly for males. Associations were maintained for maternal education when adjusting for confounders (p<0.05).

**Conclusions:**

Patterns of *HSD11B2* methylation suggest that environmental cues transmitted from the mother during gestation may program the developing fetus’s response to an adverse postnatal environment, potentially via less exposure to cortisol during development. Less methylation of placental *HSD11B2* may therefore be adaptive and promote the effective management of stress associated with social adversity in a postnatal environment.

## Introduction

There is a well-established social gradient in health whereby those experiencing the greatest socioeconomic adversity have the worst health outcomes [1]. This phenomenon is identifiable at every life stage [1]–[3] and the effects have been found to be intergenerational [4]. Moreover, socioeconomic adversity contributes to maternal morbidity during pregnancy [2] and neonatal outcomes predictive of future health risk [5]–[7]. Despite a large literature documenting the enduring health effects of parental socioeconomic adversity on offspring outcomes, the mechanisms through which adversity is transmitted between generations are not well understood.

Epigenetic alterations resulting from exposure to socioeconomic stress *in utero* may be one mechanism that explains how parental adversity affects the health of offspring. Adverse exposures experienced during pregnancy can exert life-long impacts physiologic processes in the fetus which may then influence future risk of mental and physical health conditions [8]–[10]. Epigenetic mechanisms involve alteration to the patterns of gene expression without modifying the underlying nucleotide sequence of DNA. Epigenetic mechanisms, such as DNA methylation, are particularly relevant for understanding the early origins of disease as they are sensitive to environmental exposures and can be altered during critical periods of development like gestation, and these alterations may then remain stable into adulthood [11]. While researchers are increasingly examining fetal origins hypotheses from an epigenetics perspective [9], [12], and other work has examined early life socioeconomic adversity in conjunction with DNA methylation in adulthood [13], [14], few studies have directly examined the relationship between prenatal socioeconomic adversity (as defined by occupying positions of low socioeconomic status according to income, education and occupation [15]) and DNA methylation in the offspring in the perinatal period. Therefore, we conducted one of the first studies of prenatal socioeconomic adversity and placental DNA methylation of a gene promoter sensitive to psychosocial stress [16]–[19] and predictive of future health risk [20]–[22].

The fetal environment is regulated by the placenta, which plays an active immune-endocrine functional role in pregnancy, in addition to its role in nutrient, gas, and waste exchange [20]. The placenta is also involved in the development of the child’s hypothalamic–pituitary–adrenal (HPA) axis, including the regulation of cortisol exposure to the fetus, through the actions of 11-β hydroxysteroid dehydrogenase type 2 (HSD11B2). HSD11B2 is responsible for converting cortisol into inactive cortisone, thereby protecting the fetus from exposure to deleterious stress hormones during development. However, this protective mechanism has limits; when prenatal cortisol levels are high, the protective effect of placental HSD11B2 may be diminished, thereby allowing elevated levels of glucocorticoids into fetal circulation [23]. Such overexposure to glucocorticoids is associated with low birth weight, poor infant neurodevelopment, and adulthood anxiety and cardiometabolic disorders [16], [20], [22]. While several animal and human studies suggest that prenatal stress is associated with placental HSD11B2 activity and gene expression [17], [24], [25], only one study has examined the association of prenatal stress and placental *HSD11B2* methylation. In an experimental study of gestational stress among rats, pregnant females subjected to a chronic stress exposure had increased placental DNA methylation of specific CpG sites within the *HSD11B2* gene promoter and reduced *HSD11B2* placental mRNA as compared to non-stressed controls [18]. This study suggests that stress during pregnancy may be associated with *HSD11B2* methylation, although such findings have yet to be replicated in human populations.

Environmental cues transmitted from the mother may alter fetal response to the postnatal environment [26]. Such developmental plasticity is adaptive as the child is then born into an environment it has been optimized to fit. As such, exposure to socioeconomic adversity during gestation may program the developing child to be biologically equipped to handle postnatal adversity. This may be particularly true in healthy populations subjected to everyday stressors. Animal work has shown there are dose-dependent effects of prenatal stress on multiple aspects of development where mild and severe stressors often have opposite effects in the developing brain [27]. Among humans, prenatal exposure to severe stressors or trauma is associated with increased glucortocoid receptor methylation and poor perinatal outcomes [28], [29], which may increase health risk over the life course. In situations of less severe stress like daily socioeconomic adversity, protective glucocorticoid barriers may continue to function, and the developing child may be better able to mount an adaptive biological response to manage stress in the postnatal environment. However, no study has assessed this possibility directly.

Gestational programming effects are often sex specific [30]. For example, levels of placental mRNA expression of *HSD11B2* has been shown to vary across male versus female placentas [31], and the prevalence of several physical and mental health conditions attributable in part to prenatal stress are also patterned by sex [10], [30], [32]. Differential levels of *HSD11B2* methylation by sex may help explain such observations. However, no studies have examined sex differences in methylation extent of *HSD11B2* in association with prenatal adversity.

The present study had two aims. First, we examined whether prenatal socioeconomic adversity would be associated with an adaptive epigenetic profile, namely less placental *HSD11B2* methylation. We also examined whether prenatal socioeconomic adversity and *HSD11B2* methylation associations would differ by infant sex. To achieve these aims, we examined prospective data from 444 pregnant women who delivered healthy term infants, while controlling for potential social and biological confounds. To our knowledge, this is the first study of prenatal socioeconomic adversity and *HSD11B2* methylation.

## Methods

### Ethics Statement

Study protocols were approved by the Institutional Review Boards for Women and Infants’ Hospital and Dartmouth College. Mothers provided written informed consent for participation and also for participation of her infant.

### Study Population

Study subjects are part of the ongoing Rhode Island Child Health Study, which enrolls mother and infant pairs following delivery at the Women and Infants Hospital of Rhode Island (Providence, RI, USA). Term infants (≥37 weeks) born small for gestational age (SGA, lowest 10^th^ percentile), or large for gestational age (LGA, highest 10^th^ percentile), based on birth weight and gestational age and calculated from the Fenton growth chart [33], are selected; infants appropriately sized for gestational age (AGA) matched on sex, gestational age (±3 days), and maternal age (±3 years) are also enrolled. Only singleton, viable infants are included in the study. Other exclusion criteria are maternal age (<18 or >40 years excluded), a life-threatening medical complication of the mother, and congenital or chromosomal abnormality of the infant. A structured chart review was used to collect information from the maternal inpatient medical record from delivery. While still in the hospital after delivery but prior to discharge, mothers participated in an interviewer-administered structured questionnaire to obtain information on socioeconomic adversity, health behaviors, demographics, and exposure histories. For this analysis, the first 444 participants who enrolled between September 2009 and March 2012 and had available prenatal socioeconomic adversity and placental *HSD11B2* methylation information were examined. Due to missing data on household income, derived variables that incorporate information on income (e.g., poverty, cumulative risk score) have a smaller sample size of 403. No participants from the analytic sample were exposed to synthetic glucocorticoids (e.g., prednisone, betamethasone, dexamethasone) during the prenatal period.

### Placenta Sample Collection, Nucleic Acid Extraction and Bisulfite Modification

For each subject and within 2 hours of delivery, 12 samples of placenta tissue, 3 from each of 4 quadrants (totaling approximately 8–10 g of tissue) were excised. All samples were taken from the maternal side of the placenta, 2 cm from the umbilical cord insertion site, free of maternal decidua. The samples were placed immediately in RNAlater (Life Technologies, Grand Island, NY) and stored at 4°C. At least 72 hours later, placenta samples were removed from RNAlater, blotted dry, snap-frozen in liquid nitrogen, homogenized using a mortar and pestle, and stored in sample tubes at –80°C until needed for examination. DNA was extracted from the placenta samples using the QIAmp DNA Mini Kit (Qiagen, Inc., Valencia, CA). Purified DNA was quantified using a ND-2000 spectrophotometer (Thermo Fisher Scientific Inc., Waltham, MA), and DNA samples (500 nanograms) were bisulfite-modified using the EZ DNA Methylation Kit (Zymo Research, Irvine, CA, USA.) and stored at –20°C.

### Bisulfite Pyrosequencing DNA Methylation

Pyrosequencing was performed on PCR product amplified from bisulfite-modified DNA as described previously [20] based on the region sequenced and displaying an association with expression in both choriocarcinoma cell lines and human placenta from Alikhani-Koopaei et. al [34]. In brief, the Pyromark PCR Kit (Qiagen Inc.) and the following forward and biotinylated reverse primers were used for amplification: HSD11B2-F, 5′-GGAAGTGGGGTTGTGYGTTTTTAGGTTTAAGTT -3′ and HSD11B2-R, 5′-biotin-ATACCCTTTACTAATCRCACCACC-3′ (IDT Inc., Coralville, IA). Cycling conditions were 94°C for 15 minutes followed by 45 cycles of 94°C for 30 seconds, 55°C for 1 minute and 72°C for 1 minute with a final extension of 7 minutes at 72°C. PCR products were sequenced using a PyroMark MD system and the following sequencing primer (IDT): HSD11B2-seq, 5′-GGGGTAGAGATTTTAAGAA -3′. The sequencing primer was designed to sequence four CpG sites, and the dispensation order for the assay was GTCGATGTCAGTCGTTAGTTCGTCA. The percent methylation at each CpG site was quantified using the PyroMark CpG software, version 1.0.11. (Qiagen). In order for a sample’s methylation extent to be called, it must exhibit at least a 93% bisulfite conversion rate, as assessed by pyrosequencing, and all samples examined exhibited a rate >95%. To prevent batch effects from bisulfite treatments interfering with the analysis, samples were randomized across conversion batches.

Methylation across each of the four *HSD11B2* CpG sites was averaged to obtain an overall measure of methylation. As the distribution of methylation extent was skewed, a log_10_ transformation was applied in order to approximate a normal distribution. *HSD11B2* was treated as a continuous outcome in analysis.

### Prenatal Socioeconomic Adversity

Several measures of maternal socioeconomic adversity during pregnancy were examined. Though related, indicators of socioeconomic status are not interchangeable as each may affect disease risk in different ways and have different intervention implications [15]. Therefore, measures of maternal education, poverty status, marital status and dwelling crowding were separately considered. We also examined tobacco use during pregnancy as a marker for socioeconomic adversity. Maternal education was assessed as the highest grade completed and dichotomized as low (less than high school) versus high (high school more). Poverty was assessed as whether or not a participant’s household income was at or below the Health and Human Services federal poverty line for the household size in the year of the child’s birth [35]–[38]. Marital status was dichotomized as single (never married, widowed, divorced) and married. Crowded dwelling was assessed as whether or not 7 or more people were in the household. Tobacco use during pregnancy was assessed as yes/no as recorded in the medical record.

To assess the overall risk of experiencing multiple socioeconomic adversities during pregnancy, a cumulative risk score was derived. Using composite variable construction methods common in child adversity research [39], [40], the cumulative risk score was a sum of the number of socioeconomic adversities reported by the mother (low education, living in poverty, single mother, crowded dwelling, prenatal tobacco use; range 0–5). Because no component was hypothesized to be more important than another, parts of the composite were not additionally weighted, resulting in a simple and conservative summary of the cumulative experience of socioeconomic adversity during pregnancy. Higher scores reflect greater prenatal socioeconomic adversity.

### Covariates

Maternal age, pre-pregnancy body mass index, race, infant sex and birth weight percentile were included as covariates in multivariate models based on their potential to confound prenatal socioeconomic adversity and *HSD11B2* methylation associations [41]. Race was dichotomized as white and not white based on the distribution of race/ethnicity in the sample. Pre-pregnancy body mass index (BMI) was calculated as weight in kilograms/height in meters^2^ using self-reported information. Maternal age, pre-pregnancy BMI and birth weight percentile were treated continuously.

### Statistical Analysis

First, those excluded from the analytic sample due to missing prenatal socioeconomic adversity information (n = 31) were compared to those analyzed (n = 444) in terms of maternal age, pre-pregnancy BMI, race, infant sex and birth weight percentile via χ^2^ and independent t-tests. Descriptive statistics were calculated for the analytic sample. Bivariate relations among socioeconomic factors were examined via χ^2^ tests to assess the interrelations among adversity constructs. Next, unadjusted and adjusted generalized linear models assessed the association of each socioeconomic adversity variable and cumulative risk score with methylation extent of *HSD11B2*. Sex-specific associations were assessed via stratification of adjusted linear models, and fitting interaction terms. Also, as *HSD11B2* was log_10_ transformed, coefficients were exponentiated and percent change in methylation per unit change in socioecnomic adversity were reported. Statistical significance was determined by p-values less than 0.05.

## Results

### Descriptive Statistics

The sample characteristics are listed in Table 1. Mothers were on average 29.6 years old, had an average pre-pregnancy BMI of 26.7 and 74% were white. Half of the infants were female and birth weight was on average at the 56^th^ percentile. The average methylation across CpG loci was 13.6%. Mothers experienced a range of socioeconomic adversities during pregnancy as 3% lived in a crowded dwelling, 5% smoked during pregnancy, 8% had low education, 21% lived in poverty, and 37% were single mothers. However, as would be expected in a healthy cohort, the majority of mothers reported experiencing 0 or 1 prenatal socioeconomic adversity (range 0–4). While maternal age, race, pre-pregnancy BMI, infant sex, and birth weight were not uniformly associated with socioeconomic indicators and placental *HSD11B2* methylation (data not shown), all covariates were included in multivariate analysis to be conservative. There were no significant differences between the base analytic sample and those missing socioeconomic adversity information in terms of maternal age, race, pre-pregnancy BMI, infant sex or birth weight percentile.

**Table 1:** Descriptive statistics.

Characteristic		Mean(SD)/%(n)	
*HSD11B2*, overall % methylated		13.6 (3.0)	
CpG1, % methylated		9.0 (2.9)	
CpG2, % methylated		19.5 (3.8)	
CpG3, % methylated		9.4 (2.7)	
CpG4, % methylated		16.5 (3.7)	
Maternal age, years		29.6 (5.6)	
Maternal pre-pregnancy BMI		26.7 (6.9)	
Birth weight percentile		56.2 (34.6)	
Maternal race	White	74.4 (331)	
	Not white	25.7 (114)	
Infant sex	Females	51.7 (230)	
	Males	48.3 (215)	
Maternal education	<High school	7.6 (34)	
	High school+	92.4 (411)	
Maternal poverty status	Living in poverty	21.0 (85)	
	Not living in poverty	79.0 (319)	
Maternal marital status	Single	36.9 (164)	
	Married	63.2 (281)	
Dwelling crowding	≥7 people/household	3.0 (12)	
	<7 people/household	97.3 (443)	
Prenatal tobacco use	Yes	5.2 (23)	
	No	94.8 (422)	
Cumulative risk score	0 risk factors	59.7 (241)	
	1 risk factor	19.8 (8)	
	2 risk factors	14.6 (59)	
	3 or more risk factors	5.9 (24)	

Note: n = 444; additional exclusions were made for poverty and cumulative risk score variables (n = 403).

Table 2 lists the bivariate associations among the socioeconomic adversity variables. While some factors were strongly associated in the expected directions (e.g., 84% of mothers with low education also lived in poverty), other associations are less robust (e.g., 5.9% of mothers in poverty also lived in a crowded dwelling). These results illustrate that the indicators of socioeconomic adversity are not interchangeable and should be examined independently in multivariate models.

**Table 2:** Chi-square associations among prenatal socioeconomic adversity variables.

Characteristic	Low education	Living in poverty	Single mother	Crowded dwelling	Tobacco use
	%				
Low education	--	--	--	--	--
Living in poverty	84.0***	--	--	--	--
Single mother	85.3***	77.7***	--	--	--
Crowded dwelling	14.7***	5.9*	3.6	--	--
Prenatal tobacco use	17.7***	11.8**	10.9***	0.0	--

Note: Cell entries represent the percent of individuals with the column characteristic that share the row characteristic. *p<0.05, **p<0.01, ***p<0.001.

### Prenatal Socioeconomic Adversity and Placental *HSD11B2* methylation

Table 3 lists the results from the linear regression models testing the association between prenatal socioeconomic adversity and *HSD11B2* methylation. In unadjusted models, low maternal education, prenatal tobacco use, and higher cumulative risk scores were associated with significantly lower *HSD11B2* methylation (all p<0.05). Living in poverty and single motherhood were each marginally associated with lower *HSD11B2* methylation in unadjusted models (all p<0.10). When adjusting for maternal age, pre-pregnancy BMI, race, infant sex and birth weight percentile, the association for low maternal education and *HSD11B2* methylation was maintained (p<0.05), and associations for prenatal tobacco use and cumulative risk score were attenuated to marginal significance. Associations were small to moderate in size. Low maternal education and prenatal tobacco use were each significantly associated with 8.8% less *HSD11B2* methylation compared to those with higher levels of education and not smoking during pregnancy respectively. A one unit increase in the cumulative risk score was marginally associated with 2.3% lower *HSD11B2* methylation. No associations were observed for living in poverty, single motherhood or crowded dwelling in adjusted models.

**Table 3:** Generalized linear models for the association of prenatal socioeconomic adversity and *HSD11B2* methylation.

	Unadjusted	Adjusted
Characteristic	β (SE)	
Low maternal education	–10.9 (1.0)**	–8.8 (1.0)*
Maternal poverty	–4.5 (1.0)+	–4.5 (1.0)
Single mother	–4.5 (1.0)+	–2.3 (1.0)
Crowded dwelling	–2.3 (1.1)	–2.3 (1.1)
Prenatal tobacco use	–8.8 (1.0)*	–8.8 (1.0)+
Cumulative risk score	–2.3 (1.0)*	–2.3 (1.0)+

Note: Each socioeconomic factor was modeled as the independent variable associated with *HSD11B2* methylation extent as the dependent variable. Beta coefficents represent percent change in methylation per unit difference in socioeconomic adversity. Adjusted models control for maternal age, pre-pregnancy BMI, race, infant sex and birth weight percentile. n = 444; additional exclusions were made for poverty and cumulative risk score variables (n = 403).

+ p<0.10, *p<0.05, **p<0.01.

### Sex Specific Associations

When stratifying adjusted models by infant sex, some differences in prenatal socioeconomic adversity and placental *HSD11B2* methylation findings were observed suggesting associations may be more robust for males than for females (Table 4). Low maternal education, living in poverty and higher cumulative risk scores were associated with significantly less *HSD11B2* methylation for males but not for females (all p<0.05). Associations were again small to moderate in size. Compared to those experiencing low levels of prenatal socioencomic adversity, low maternal education, living in poverty during pregnancy and a one unit increase in cumulative risk scores were each associated with 12.9%, 8.8% and 4.5% lower *HSD11B2* methylation among males respectively. However, the analogous interaction terms for these associations were not significant (β_low education_ =  –0.05, SE =  1.0, p =  0.89; β_poverty_  =  –2.3, SE = 1.0, p =  0.73; β_cumulative risk_ =  –0.46, SE = 1.0, p = 0.87).

**Table 4:** Sex stratified, adjusted models for the association of prenatal socioeconomic adversity and *HSD11B2* methylation.

	Females	Males
Characteristic	β (SE)	
Low maternal education	–6.7 (1.0)	–12.9 (1.1)*
Maternal poverty	–0.46 (1.0)	–8.8 (1.0)*
Single mother	–0.12 (1.0)	–4.5 (1.0)
Crowded dwelling	–6.7 (1.1)	–0.69 (1.1)
Prenatal tobacco use	–8.8 (1.1)	–8.8 (1.1)
Cumulative risk score	–1.1 (1.0)	–4.5 (1.0)*

Note: Each socioeconomic factor was modeled as the independent variable associated with *HSD11B2* methylation extent as the dependent variable. Beta coefficents represent percent change in methylation per unit difference in socioeconomic adversity. These adjusted models control for maternal age, pre-pregnancy BMI, race, and birth weight percentile. n = 444; additional exclusions were made for poverty and cumulative risk score variables (n = 403).

* p<0.05.

## Discussion

This study demonstrated that prenatal socioeconomic adversity was associated with lower levels of placental *HSD11B2* methylation, particularly for males. Patterns of methylation were consistent with a developmental origins of disease framework whereby environmental cues transmitted from the mother during gestation may have programmed the developing fetus’s response to an adverse postnatal environment. Previous work by our group found *HSD11B2* expression to be moderately and negatively correlated with methylation extent in this sample [20]. Less methylation of placental *HSD11B2* may therefore be adaptive and promote the effective management of stress associated with social adversity. Moreover, effect sizes were small to moderate in size and similar to what has been observed in other studies of placental *HSD11B2* and infant outcomes [20]. These findings are particularly noteworthy as this study was the first to examine prenatal socioeconomic adversity and placental DNA methylation of a gene promoter sensitive to psychosocial stress [16]–[19] and predictive of future health risk [20]–[22].

While congruent with a developmental origins of disease framework, the direction of effects we observed is not consistent with findings from the other published study in this area. Peña et al [18] found prenatal stress to be associated with increased placental *HSD11B2* methylation in sample of rats. Several methodological differences may help explain such inconsistencies across the two studies. We conducted an observational study among 444 humans using naturally varying levels of maternal socioeconomic adversity as indicative of prenatal stress. Peña et al employed an experimental design with 12 rats (6 test subjects, 6 controls) and used forced restraint during gestation to induce stress. Epigenetic responses to stress may manifest differently in humans versus animals and vary according to level and type of stress exposure. Moreover, our large sample may have been better powered to detect epigenetic changes related to stress common in a healthy population. As this is an emerging area of research, more work is needed to better characterize the association of prenatal stress and placental *HSD11B2* methylation.

In adjusted models, maternal education was the only prenatal socioeconomic factor significantly associated with *HSD11B2* methylation, with marginal associations evident for tobacco use and the cumulative risk score. This is somewhat surprising given the bivariate associations among socioeconomic indicators. However, low maternal education, defined as having a less than high school education, reflects a stable socioeconomic factor likely established before childbearing. Conversely, poverty, residential crowding and marital status are more changeable over time and may not capture an accumulated socioeconomic burden that may be relevant for placental *HSD11B2* methylation. As such, these findings underscore the utility for examining multiple socioeconomic factors as independent predictors as each may have different relationships with methylation, different linking mechanisms to disease and implications for intervention [15].

We found some evidence that the programming effects of prenatal socioeconomic adversity on placental *HSD11B2* methylation may be sex-specific. Adversity was significantly associated with less *HSD11B2* methylation for males but not females in sex-stratified models, although interaction terms were not significant. This finding is consistent with the observed male-dominated prevalence of some cardiometabolic diseases that have developmental origins [30] and suggests that males may be more sensitive to intrauterine exposure than females. As this is an emerging area of research, we present this association for descriptive purposes and encourage others to identify mechanisms (e.g., interactions with sex hormones) that could help explain sex-patterning in prenatal adversity and *HSD11B2* methylation associations.

In addition to traditional indicators of socioeconomic adversity like low maternal education and poverty status, we also examined tobacco use during pregnancy as a marker for prenatal adversity. While not a measure of socioeconomic status per se [15], smoking during pregnancy is associated with prenatal socioeconomic status and poor perinatal outcomes [7], and other work has found other forms of prenatal substance use to function as an intrauterine stressor and alter the expression genes involved in the HPA axis [25]. Tobacco use is also a known contributing factor to DNA methylation [42]. In this study, prenatal tobacco use was modestly associated with multiple markers of socioeconomic adversity in the expected directions (e.g., low education associated with greater likelihood of prenatal tobacco use), and patterns of methylation were similar for the traditional socioeconomic variables and prenatal tobacco use (e.g., greater adversity, less *HSD11B2* methylation). This consistency of findings for prenatal tobacco use and other socioeconomic markers help to validate the socioeconomic adversity and methylation findings.

Although our results suggest that prenatal exposure to stress may yield an adaptive epigenetic profile at birth, this profile may be altered in an adverse postnatal environment as programming continues through infancy [43]. For example, in cross-fostering studies of rats where biological offspring of low-licking and grooming dams (low maternal care) are reared by high-licking and grooming dams (high maternal care) and vice versa, Weaver and colleagues demonstrated that the programming effects of postnatal maternal care occur independently from genomic influences, and that epigenetic mechanisms may mediate the effect of maternal care on glucocorticoid expression [44]. Among humans, socioeconomic adversity is also associated with maternal parenting behaviors: low socioeconomic status parents are more likely to use harsh and inconsistent discipline with their children whereas higher socioeconomic status parents are more likely to exhibit warm and empathetic parenting behaviors [45], [46]. Such variations in parental care is predictive of offspring emotional and physical health years later [47], [48]. These studies indicate that programming is dynamic; prenatal factors are not the sole determinants of epigenetics risk or resilience factors. While our study shows that an infant may be born to handle a certain degree of stress in the postnatal environment, such adaptive capacities may be eventually overrun through an accumulation of risks stemming from socioeconomic adversity. We encourage future work to examine how epigenetic profiles identifiable at birth may be modified by later socioeconomic adversity.

This study has some limitations. First, we are limited in our ability to conclusively define the mechanism linking the intrauterine environment with epigenetic modulation. While socioeconomic adversity is known to induce significant psychosocial stress [49], [50], we did not explicitly measure prenatal stress and cannot quantify the level of stress experienced by mothers during pregnancy. We suggest that future work examine self-reported and physiologic measures of maternal stress as mechanisms linking prenatal socioeconomic adversity to placental *HSD11B2* methylation. Moreover, this study does not consider placental *HSD11B2* methylation in association with infant outcomes. While it was our intent to demonstrate epigenetic programming effects of prenatal socioeconomic adversity, the clinical implications of this work are not known. Previous work in our sample has found placental *HSD11B2* methylation to be associated with infant neurodevelopment and birth weight [20]. We encourage future work to link markers of prenatal adversity, *HSD11B2* methylation with infant health outcomes. Finally, in restricting our analysis to include only healthy pregnancies, we may be constraining the variance of the exposure variables as socioeconomic adversity is associated with poor perinatal outcomes [7].

These limitations notwithstanding, this study has a number of strengths. This study represents one of the first to consider the association of prenatal adversity and placental *HSD11B2* methylation in humans. We focused on a healthy population of infants from uncomplicated pregnancies, thereby mitigating concerns that associations could be confounded by maternal or infant illness. Moreover, the sample is large and well powered to detect small to moderate effects of prenatal socioeconomic adversity. Also, where other work has examined the epigenetic effects of trauma and severe stress during pregnancy [28], we examined moderate levels of adversity that may be more common in the general population. Finally, we examined multiple measures of prenatal SES, which allows for a broad examination of the role prenatal socioeconomic adversity plays in the epigenetic modulation of *HSD11B2*.

Evidence is accumulating that prenatal adversity contributes to epigenetic alterations of offspring. Our study adds to this emerging evidence base and demonstrated that prenatal socioeconomic adversity was associated with less placental *HSD11B2* methylation, particularly for males. Findings were consistent with a developmental origins of disease framework whereby the developing fetus has been programmed to “expect”, and is thus prepared to effectively manage, adversity in the postnatal environment. This study suggests that the plasticity of fetal development can potentially protect the offspring from deleterious prenatal exposures. Postnatal experiences will help determine whether or not such safeguards can last for a lifetime.
